# Pathological mutations in PNKP trigger defects in DNA single-strand break repair but not DNA double-strand break repair

**DOI:** 10.1093/nar/gkaa489

**Published:** 2020-06-06

**Authors:** Ilona Kalasova, Richard Hailstone, Janin Bublitz, Jovel Bogantes, Winfried Hofmann, Alejandro Leal, Hana Hanzlikova, Keith W Caldecott

**Affiliations:** Department of Genome Dynamics, Institute of Molecular Genetics of the Czech Academy of Sciences, Prague 4, 142 20, Czech Republic; Genome Damage and Stability Centre, University of Sussex, Falmer, Brighton BN1 9RQ, UK; Department of Human Genetics, Hannover Medical School, Hannover, Germany; Servicio de Cirugía Reconstructiva, Hospital Rafael Ángel Calderón Guardia, Caja Costarricense de Seguro Social, San José, Costa Rica; Department of Human Genetics, Hannover Medical School, Hannover, Germany; Section of Genetics and Biotechnology, School of Biology, University of Costa Rica, San José, Costa Rica; Department of Genome Dynamics, Institute of Molecular Genetics of the Czech Academy of Sciences, Prague 4, 142 20, Czech Republic; Genome Damage and Stability Centre, University of Sussex, Falmer, Brighton BN1 9RQ, UK; Department of Genome Dynamics, Institute of Molecular Genetics of the Czech Academy of Sciences, Prague 4, 142 20, Czech Republic; Genome Damage and Stability Centre, University of Sussex, Falmer, Brighton BN1 9RQ, UK

## Abstract

Hereditary mutations in polynucleotide kinase-phosphatase (PNKP) result in a spectrum of neurological pathologies ranging from neurodevelopmental dysfunction in *microcephaly with early onset seizures* (MCSZ) to neurodegeneration in *ataxia oculomotor apraxia-4* (AOA4) and *Charcot-Marie-Tooth disease* (CMT2B2). Consistent with this, PNKP is implicated in the repair of both DNA single-strand breaks (SSBs) and DNA double-strand breaks (DSBs); lesions that can trigger neurodegeneration and neurodevelopmental dysfunction, respectively. Surprisingly, however, we did not detect a significant defect in DSB repair (DSBR) in primary fibroblasts from PNKP patients spanning the spectrum of PNKP-mutated pathologies. In contrast, the rate of SSB repair (SSBR) is markedly reduced. Moreover, we show that the restoration of SSBR in patient fibroblasts collectively requires both the DNA kinase and DNA phosphatase activities of PNKP, and the fork-head associated (FHA) domain that interacts with the SSBR protein, XRCC1. Notably, however, the two enzymatic activities of PNKP appear to affect different aspects of disease pathology, with reduced DNA phosphatase activity correlating with neurodevelopmental dysfunction and reduced DNA kinase activity correlating with neurodegeneration. In summary, these data implicate reduced rates of SSBR, not DSBR, as the source of both neurodevelopmental and neurodegenerative pathology in PNKP-mutated disease, and the extent and nature of this reduction as the primary determinant of disease severity.

## INTRODUCTION

DNA strand breaks can arise endogenously or can result from exogenous sources of DNA damage such as ionizing radiation and chemical genotoxins. To combat the genotoxic impact of DNA damage, cells have evolved multiple biochemical pathways to detect and repair DNA strand breaks (1,2). Importantly, defects in DNA strand break repair can result in a variety of different disease pathologies, highlighting the threat posed by DNA breaks to human health (3–5). For example, microcephaly and developmental delay are often present in individuals with defects in non-homologous end-joining (NHEJ); one of the two major pathways by which DNA double-strand breaks (DSBs) are repaired (4). In contrast, the primary pathology resulting from defects in the repair of DNA single-strand breaks (SSBs) is neurodegeneration, and in particular progressive cerebellar ataxia (5,6). Examples of the latter are individuals with *spinocerebellar ataxia with axonal neuropathy 1* (SCAN1), *ataxia with oculomotor apraxia type 1* (AOA1), and *ataxia with oculomotor apraxia-XRCC1* (AOA-XRCC1), in which the DNA strand break repair proteins TDP1, aprataxin, and XRCC1 are mutated, respectively (7–10).

Arguably one of the most common sources of neurological disease associated with defects in DNA strand break repair are mutations in the enzyme polynucleotide kinase-phosphatase (PNKP) (11). PNKP possesses both DNA 5′-kinase and DNA 3′-phosphatase activity and thereby can convert 5′-hydroxyl and 3′-phosphate termini to the canonical 5′-phosphate and 3′-hydroxyl moieties necessary for completion of DNA strand break repair (12,13). Whereas 3′-phosphate termini are present at ∼70% of DNA breaks induced by reactive oxygen species and ionising radiation, both 3′-phosphate and 5′-hydroxyl termini are generated at DNA strand breaks induced by the abortive activity of topoisomerase 1 (TOP1) (14,15). Notably, PNKP interacts with protein complexes involved in the repair of SSBs and DSBs in mammalian cells via the interaction of its amino-terminal FHA domain with the SSBR and DSBR scaffold proteins XRCC1 (16–18) and XRCC4 (19–22), respectively.

Mutations in PNKP result in three clinically distinct neurological diseases. The most severe of these is *microcephaly with early onset seizures* (MCSZ), a neurodevelopmental disease associated with microcephaly, early-onset seizures and developmental delay (23). In addition, PNKP mutations are the cause of the neurodegenerative disease *ataxia with oculomotor apraxia 4* (AOA4), which exhibits progressive cerebellar atrophy and ataxia oculomotor apraxia (24–26). Strikingly, some affected individuals possess elements of both of these diseases; presenting with both microcephaly and progressive cerebellar atrophy (26–30). Finally, PNKP mutations were also identified recently in *Charcot-Marie-Tooth disease 2B2* (CMT2B2), which is associated with mild axonal peripheral polyneuropathy and relatively late-onset cerebellar ataxia (31–33).

Here, we have addressed the relationship between defects in SSBR, DSBR, and neurological pathology in patient-derived cells from individuals with mutations in PNKP spanning the spectrum of PNKP-associated pathologies. Surprisingly, our data implicate unrepaired SSBs as the source of both the neurodevelopmental and neurodegenerative pathology in PNKP-associated disease, and the level of residual SSBR as the primary determinant of disease severity. Our data also implicate the two activities of PNKP in different aspects of the disease pathology; with reduced DNA phosphatase and DNA kinase activity correlating best with neurodevelopmental dysfunction and neurodegeneration, respectively.

## MATERIALS AND METHODS

### Cell lines

The control human primary fibroblasts 1BR3 (denoted here as 1BR) and the PNKP patient-derived primary fibroblasts AOA4/MCSZ (7,26), MCSZ-I (34), CMT2B2-(I-V) (32) and XRCC1 patient-derived primary fibroblasts (7) were grown in Minimum Essential Media (MEM, Gibco) supplemented with 15% fetal bovine serum, 2 mM glutamine and the antibiotics penicillin (100 units/ml) and streptomycin (100 μg/ml). Lymphoblastoid cells (LCLs) derived from CMT2B2 patients with a pathogenic heterozygous mutation (p.Y145S) in myelin protein zero (denoted here CMT-control), PNKP patients CMT2B2-(I-VI) (32), AOA4-I (25), AOA4-II and father and mother parental controls (35), and ‘WT’ control LCLs were grown in RPMI-1640 Media (Sigma) supplemented with 10% fetal bovine serum and the antibiotics as above in a humidified atmosphere of 5% CO_2_ at low oxygen (5%) at 37°C.

### siRNA transfection

Where indicated, cells were transfected with mix of either PNKP siRNA #1: 5′-CCGGAUAUGUCCACGUGAA-3′ and PNKP siRNA #2: 5′-GGAAACGGGUCGCCAUCGA-3′ or non-target siRNA #1: 5′-UGGUUUACAUGUCGACUAA-3′ and non-target siRNA #2: 5′-UGGUUUACAUGUUGUGUGA-3′) using Lipofectamine RNAiMAX (Life Technologies) 48–72 h before experiment.

### Constructs, protein purification and transfection

Wild-type recombinant human PNKP (PNKP^WT^) and PNKP with a mutated FHA domain (R35A; PNKP^FHA^) or catalytically inactive phosphatase domain (D171N; PNKP^PD^) harboured an N-terminal His tag if expressed from the bacterial expression construct pET16b or N-terminal EYFP if expressed from the mammalian expression construct pEYFP-C1. PNKP proteins were expressed in *E.coli* from pET16b and purified by metal-chelate affinity chromatography and gel filtration. For transfection of human cells with purified recombinant PNKP, 2 μg of control bovine serum albumin (BSA) or the indicated PNKP protein was electroporated into 2×10^5^ primary human fibroblasts using a NEON Transfection System (Invitrogen) according to the manufacturer's instructions. Cells were employed for experiments 18 h post-transfection. For transfection with mammalian expression constructs, 1 μg of control pEYFP-C1 or pEYFP-C1 encoding wild type or the indicated mutant PNKP were electroporated into 2×10^5^ primary human fibroblasts as described above. Transfected cells were employed in experiments 48 h post-transfection.

### Antibodies and western blotting

Primary antibodies were anti-pan-ADP-ribose binding reagent (MABE1016, Millipore), anti-PNKP N-terminal (ab170954, Abcam), anti-PNKP C-terminal (ab18107, Abcam), anti-β actin (66009, Proteintech), anti-γH2AX (9718, Cell Signaling), and anti-PCNA (sc-56, Santa Cruz Biotechnology). Secondary antibodies for western blotting were horseradish peroxidase (HRP)-conjugated goat anti-rabbit (170-6515, Bio-Rad) and goat anti-mouse (170-6516, Bio-Rad) and for indirect immunofluorescence were donkey anti-rabbit Alexa 488 (A21206, Invitrogen) and donkey anti-mouse Alexa 647 (A-31571, Invitrogen). Samples for western blotting were lysed in SDS sample buffer and subjected to SDS-PAGE, transferred onto nitrocellulose membrane and detected by the appropriate specific primary and HRP-conjugated secondary antibodies.

### Alkaline comet assays

The level of DNA strand breaks was evaluated by alkaline comet assays following DMSO treatment or treatment with 10 μM CPT for 60 min at 37°C or following irradiation with 20 Gy of X-rays (X-RAD 225XL, Accella) with indicated time of recovery. The average comet tail moment in 100 cells per sample was evaluated by Comet Assay IV software (Perceptive Instruments).

### Indirect immunofluorescence microscopy

For indirect immunofluorescence microscopy, cells were cultured and treated where indicated with 10 μM CPT (C9911, Sigma) for 45 min, irradiated (X-RAD 225XL, Accella) with 20 Gy of X-rays on ice or 2 Gy of X-rays at RT in the presence of DMSO (D2650, Sigma), 10 μM PARG inhibitor (PDD 0017273; 5952, Tocris Bioscience) or 5 μM DNA-PK inhibitor (NU 7441; 3712, Tocris Bioscience). Cells were fixed with 4% formaldehyde and immunostained as described previously (7). Images were taken using a DMi6000 microscope (Leica) with 40× dry objective. Automated wide-field microscopy was performed on scanR system (Olympus) with scanR Image Acquisition and Analysis Software, 40 x/0.95NA (UPLSAPO 2 40×) dry objective.

### DSBR assays

To measure DSBR, cells were pre-extracted with 0.2% Triton-X 100 for 2 min on ice prior fixation. Cells were stained for PCNA, γH2AX, and counterstained for DNA using 1 μg/ml DAPI (202710100, Acros). The cell cycle phase was determined based on the presence of PCNA signal and the DNA content of individual cell nuclei. PCNA-positive cells were gated as S phase cells, and the DNA content of PCNA-negative cells was compared to gate cells in G1 phase or G2 phase . Cells that could not be gated based on these criteria were excluded from the analysis.

### PNKP biochemical activity

PNKP substrate was prepared by annealing equimolar amounts of fluorophore-labeled deoxyriboligonucleotides (Midland Certified Reagent Company). To measure both 3′-phosphatase and 5′-kinase activities, oligonucleotides with 3′-phosphate ‘S1’ [5′-(TAMRA)-TAGCATCGATCAGTCCTC-3′-P] and 5′-hydroxyl ‘S2’ [5′-OH-GAGGTCTAGCATCGTTAGTCA-(6-FAM)-3′] were annealed to a complementary strand oligonucleotide [5′-TGACTAACGATGCTAGACCTCTGAGGACTGATCGATGCTA-3′] in annealing buffer (10 mM Tris pH 7.5, 200 mM NaCl, 1 mM EDTA) (36). To test only 3′-phosphatase activity, ‘S1’ was used in a combination with ‘C2’ [5′-P-GAGGTCTAGCATCGTTAGTCA-(6-FAM)-3′] and to test only 5′-kinase activity, ‘S2’ was used in a combination with ‘C1’ [5′-(TAMRA)-TAGCATCGATCAGTCCTC-3′-OH]. Cell-free protein extracts were prepared in lysis buffer [25 mM Tris, pH 7.5, 10 mM EDTA, 10 mM EGTA, 100 mM NaCl, 1 % Triton X-100, cOmplete protease inhibitors (Roche)], incubated on ice for 15 min and centrifuged at 16 000 × g, 20 min at 4°C. The indicated amounts of purified PNKP proteins or cell-free protein extracts were incubated with 50 nM substrate and 1 μM single-stranded nuclease competitor oligonucleotide [5′-AAAGATCACAAGCATAAAGAGACAGG-3′] in reaction buffer (25 mM Tris, pH 7.5, 130 mM KCl, 10 mM MgCl_2_, 1 mM DTT, 1 mM ATP) at 37°C for 10 or 60 min. 50 μl reactions were terminated by addition of 50 μl quenching buffer (90% formamide, 50 mM EDTA, 0.006% Orange G). 10 μl of each reaction was separated on a 20% denaturing polyacrylamide gel and analyzed on a PharosFX Molecular Imager System (Bio-Rad).

## RESULTS

### Reduced PNKP protein and activity in PNKP patient-derived fibroblasts

**Figure 1.**
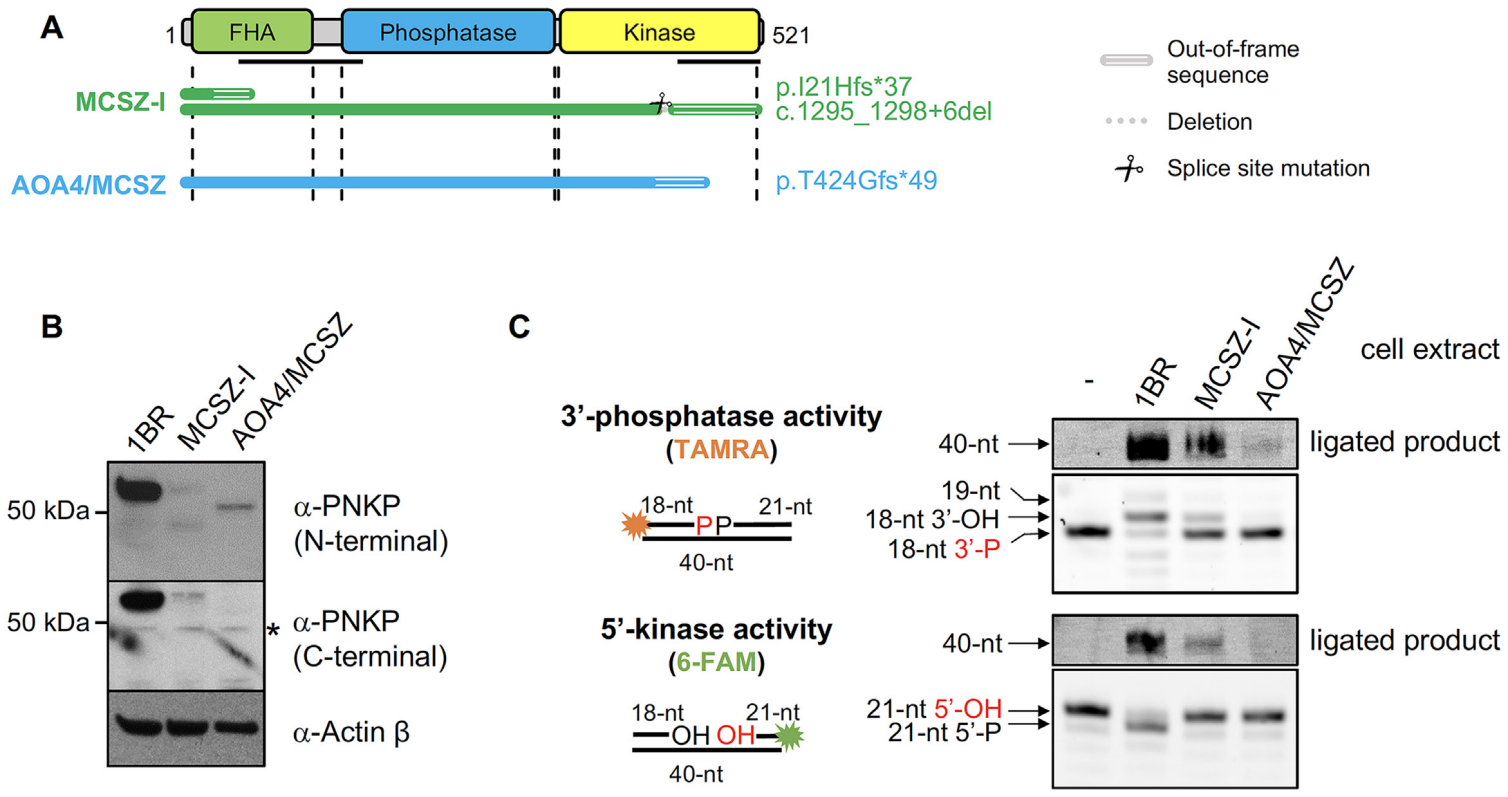
Reduced PNKP protein and activity in PNKP patient-derived fibroblasts. (**A**) Cartoon showing PNKP functional domains and the mutations present in the patient-derived fibroblast cell lines MCSZ-I and AOA4/MCSZ. The former is compound heterozygous for two frame-shift mutations and the latter is homozygous for a single frame-shift mutation. The location of the epitopes detected by the N- and C-terminal antibodies employed in this study are indicated *(black horizontal lines)*. (**B**) PNKP protein levels in control patient-derived fibroblasts from an unaffected individual (1BR) and the PNKP patient-derived fibroblasts MCSZ-I and AOA4/MCSZ, measured by western blotting using N- and C-terminal specific anti-PNKP antibodies. *Black asterisk* denotes an unspecific band. β actin was employed as a loading control. (**C**) PNKP activity in control (1BR) and PNKP patient-derived (MCSZ-I, AOA4/MCSZ) fibroblast cell extracts. TAMRA- or 6-FAM-labelled oligonucleotide duplex harbouring a SSB with a 3′-phosphate or 5′-hydroxyl terminus, respectively, was incubated with the indicated cell extracts (30 μg total protein) for 10 min (to measure kinase and phosphatase activity) or 60 min (to measure ligated product) at 37°C prior to fractionation by denaturing PAGE. *Arrows* indicate the positions of the TAMRA- labelled 3′-phosphatase substrate (‘18-nt 3′-P’), 6-FAM-labelled 5′-kinase substrate (‘21-nt 5′-OH’), and intermediates of their repair resulting from 3′-phosphatase activity (‘18-nt 3′-OH’), 5′-kinase activity (‘21-nt 5′-P’), DNA gap filling (‘19-mer’), and DNA ligation (‘40-nt’).

To identify the DNA strand break repair defects in PNKP-associated neurological disease we initially employed primary human fibroblasts derived from two PNKP-mutated individuals spanning the spectrum of PNKP-related disease pathologies and, as a control, primary human fibroblasts derived from an unaffected individual (1BR). One of the affected individuals (MCSZ-I) is a patient with MCSZ and is a compound heterozygote harbouring a 1-bp duplication in the FHA domain in one allele (c.63dupC) and a 10-bp deletion spanning the exon14/intron14 splice site in the DNA kinase domain in the second allele (c.1295_1298) (Figure 1A) (34). Both of these mutations are predicted to result in translational frameshifts in, or upstream of, the DNA 5′-kinase domain. The second affected individual is a patient with combined AOA4/MCSZ and is homozygous for a 17-bp duplication in exon 14 (c.1250_1266dup), which again results in a translational frameshift within the DNA kinase domain (Figure 1A) (26).

Both MCSZ-I and AOA4/MCSZ cells possess a very small amount of residual PNKP protein, as measured by western blotting (Figure 1B). The residual PNKP in AOA4/MCSZ cells migrated faster than wild type PNKP and was detected only by an N-terminal-specific antibody, consistent with both PNKP alleles in this cell line encoding a severely truncated 5′-kinase domain. In contrast, the residual PNKP in MCSZ-I cells migrated at two positions; one that was close to full-length PNKP and detected by both N- and C-terminal antibodies and one that was severely truncated and detected by N-terminal antibody. AOA4/MCSZ cells lacked detectable 5′-kinase activity, consistent with the truncated DNA kinase domain, but did possess a small amount of residual 3′-phosphatase activity, as measured by the appearance of a small amount of fully repaired (ligated) 3′-phosphate oligonucleotide substrate (Figure 1C). In contrast, consistent with our previous report (34), MCSZ-I cells exhibited significant levels of both DNA 3′-phosphatase and 5′-kinase activity (Figure 1C). The residual 5′-kinase activity in this cell line is most likely encoded by the small amount of near-full length PNKP polypeptide detected in this cell line, resulting from alternative splicing and/or translation-initiation downstream of the mutated FHA domain (34).

### Normal rates of DSBR in PNKP patient-derived fibroblasts

**Figure 2.**
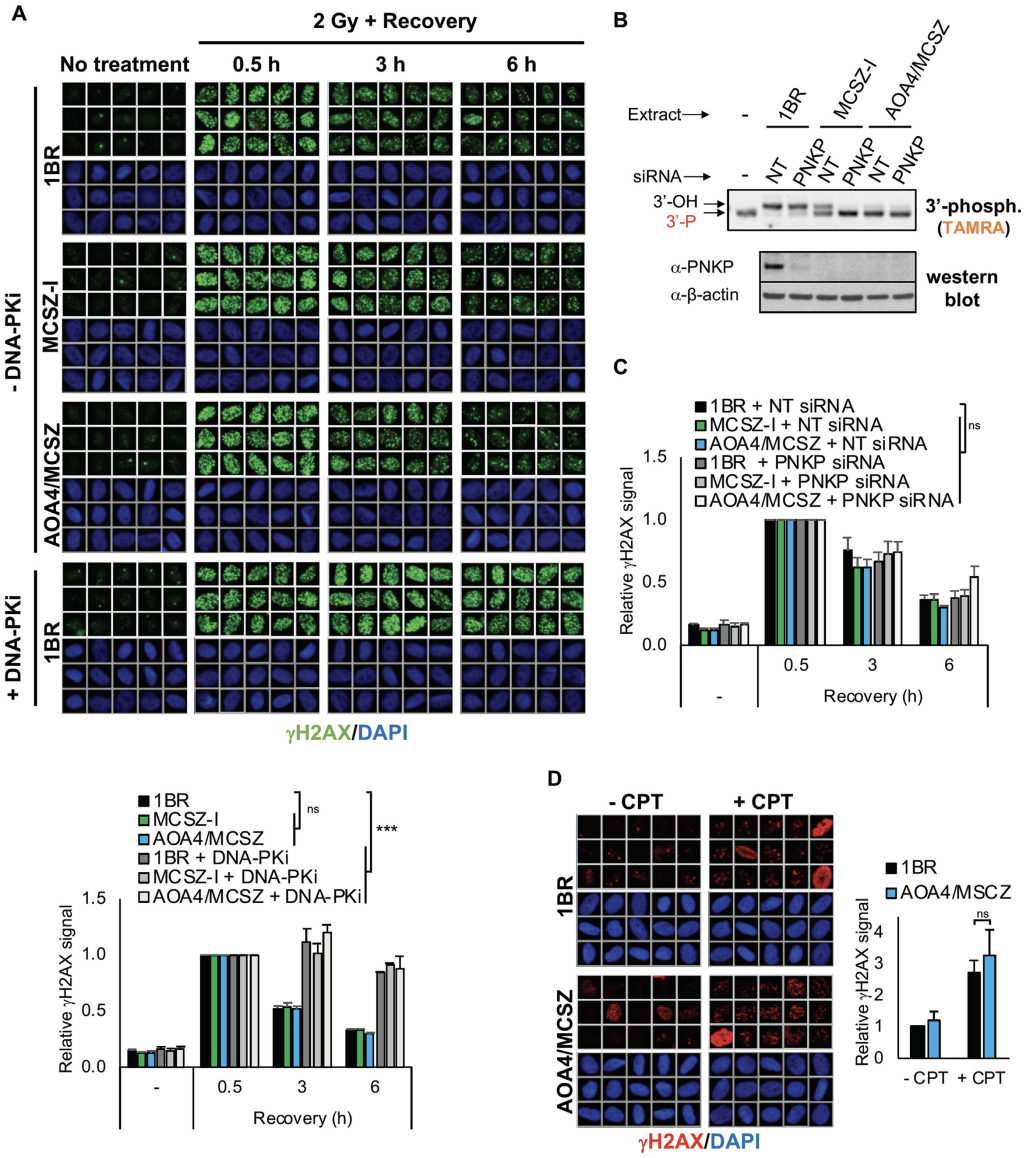
A normal rate of DSBR in PNKP patient-derived fibroblasts. (**A**) Representative scanR images (*top*) and quantification (*bottom*) of γH2AX in control (1BR) and PNKP patient-derived (MCSZ-I and AOA4/MCSZ) fibroblasts before and at the indicated times after ionizing radiation (2 Gy) in the absence or presence of 5 μM DNA-PK inhibitor (DNA-PKi was added 1 h before irradiation). Cell cycle populations were gated according to PCNA positivity (S phase) and DNA content (G1 and G2) by DAPI staining. Only the data for G1 cells are shown. For quantification, data are normalized to the 0.5 h time point and are the mean (±SEM) of three independent experiments. Statistical significance was determined by two-way ANOVA (*ns*, not significant; ***P < 0.001). (**B**) DNA 3′-phosphatase activity and western blotting of cellular extracts from control 1BR and PNKP patient fibroblasts (MCSZ-I and AOA4/MCSZ) transfected with non-target (NT) or *PNKP* siRNA. Oligonucleotide duplex substrate containing a SSB with a 3'-phosphate terminus was incubated with cellular extracts for 10 min. (**C**) Quantification of γH2AX in the indicated cell lines before and at the different times after ionizing radiation (2 Gy). Cells were transfected with non-targeting siRNA (NT) or *PNKP* siRNA 48–72 h prior to irradiation. Quantification and statistics were conducted as described in (A). Representative scanR images are shown in Supplementary Figure S1C. (**D**) Representative scanR images (*left*) and quantification (*right*) of γH2AX in the indicated cell lines after a 45 min incubation with DMSO vehicle or 10 μM camptothecin (CPT). Data are normalized to DMSO-treated control (1BR) cells and are the mean (±SEM) of three independent experiments. Statistical significance was determined by one-tailed *t*-test (*ns*, not significant).

Defects in the repair of DSBs by NHEJ resulting from hereditary mutations in XRCC4, DNA ligase IV, or Cernunnos/XLF are strongly implicated in neurodevelopmental/microcephalic pathologies similar to those observed in individuals with mutations in PNKP (37–41). Consequently, because PNKP has been shown to be involved in NHEJ (19,20,42), it seemed plausible that unrepaired DSBs might contribute to PNKP-mutated disease. To address this possibility, we employed scanR high-content imaging to quantify the level of γH2AX, an established and sensitive marker of DSBs (43,44), in PNKP patient fibroblasts following treatment with ionizing radiation (IR) or camptothecin (CPT); physiologically relevant sources of DSBs that are substrates for PNKP. γH2AX levels rapidly increased in 1BR control fibroblasts within 30 min following IR, and declined to nearly background levels over a subsequent 6 h repair period (Figure 2A and Supplementary Figure S1A). Surprisingly, however, the rate at which levels of IR-induced γH2AX declined in MCSZ and AOA4/MCSZ fibroblasts was similar to 1BR, suggesting that the rate of DSBR in PNKP patient-derived fibroblasts was largely normal. That these experiments measured NHEJ was ensured by quantifying γH2AX only in cells in G1-phase of the cell cycle at the time of analysis, which based on their cell cycle profile (Supplementary Figure S1B) were primarily in G1 or G2 (>80%) at the time of irradiation; cell cycle phases during which NHEJ is the primary determinant of DSBR proficiency (45). Indeed, treatment of wild type and PNKP patient fibroblasts with an inhibitor of the NHEJ enzyme DNA-dependent protein kinase (DNA-PKi) resulted, as expected, in a profound defect in DSBR (Figure 2A and Supplementary Figure S1A).

To examine whether NHEJ proficiency in the PNKP patient-derived fibroblasts reflected the presence of residual PNKP activity we transfected the cells with *PNKP* siRNA (Figure 2B). However, while we detected a small increase in the persistence of γH2AX in one of the two PNKP patient-derived cell lines (AOA4/MCSZ) following IR, this increase was not statistically significant (Figure 2C and Supplementary Figure S1C). The absence of a detectable defect in DSBR was not restricted to DSBs induced by IR, because we also failed to detect any significant difference in the accumulation of γH2AX between 1BR control and AOA4/MCSZ patient fibroblasts following treatment with camptothecin (CPT) (Figure 2D), which promotes abortive TOP1 activity and induces DNA breaks possessing 3′-phosphate and 5′-hydroxyl termini that are substrates for both activities of PNKP (15,46). Collectively, these results suggest that a defect in DSBR is not a cause of the neuropathology that is associated with PNKP-mutated diseases.

### Reduced Rates of SSBR in PNKP Patient-Derived Fibroblasts

**Figure 3.**
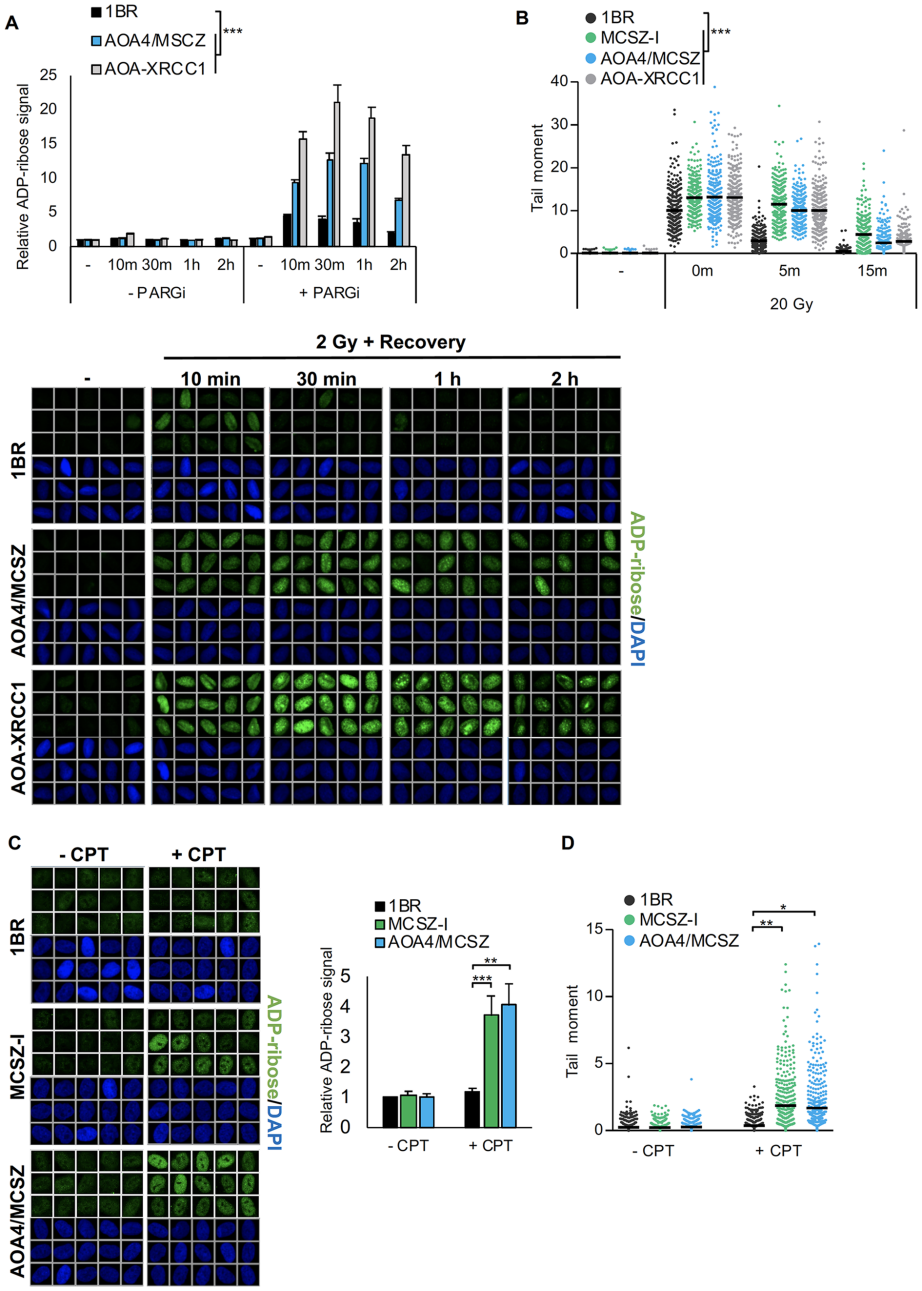
A reduced rate of SSBR in PNKP patient-derived fibroblasts. (**A**) DNA single-strand breaks quantified by measuring nuclear ADP-ribose levels in control (1BR) and PNKP patient-derived (AOA4/MCSZ) or XRCC1 patient-derived (AOA-XRCC1) fibroblasts before and at the indicated times after treatment with ionizing radiation (2 Gy). Where indicated, cells were incubated in the presence of 10 μM PARG inhibitor (PARGi) 30 min prior to and after IR to suppress poly(ADP-ribose) degradation. Data were quantified by scanR high-content imaging and are the mean (±SEM) of three independent experiments, with statistical significance determined by two-way ANOVA (***P < 0.001). Representative scanR images of single cell galleries (from samples employing PARG inhibitor) are also shown (*bottom*). (**B**) DNA strand breaks quantified by alkaline comet assays in the indicated primary fibroblasts before and at the indicated times after ionizing radiation (20 Gy). Data are the individual comet tail moments of 300 cells per sample combined from three independent experiments. The horizontal bars show the mean tail moment of the 300 cells. Statistically significant differences were determined using the individual means from the three independent experiments (100 cells/sample) by two-way ANOVA (***P < 0.001). (**C**) DNA strand breaks quantified by measuring nuclear ADP-ribose levels in control (1BR) and PNKP patient-derived (MCSZ-I & AOA4/MCSZ) fibroblasts after incubation for 45 min with DMSO vehicle or with 10 μM camptothecin (CPT). Representative scanR images (*left*) and quantification (*right*) are shown. Data are normalized to DMSO-treated 1BR cells and are the mean (±SEM) of seven independent experiments. Statistical analysis (one-tailed *t*-test) is indicated (***P < 0.001; **P < 0.01). (**D**) DNA strand breaks quantified by alkaline comet assays in the indicated cell lines treated with DMSO vehicle or with 10 μM CPT for 60 min. Data are as described in (*B)*. Statistical significance was determined by one-tailed *t*-test (**P < 0.01; *P < 0.05).

Next, we examined the rate of SSBR in the PNKP patient-derived fibroblasts by quantifying the level of nuclear poly(ADP-ribose) in cells following DNA damage, in the presence of an inhibitor of poly(ADP-ribose) glycohydrolase (PARG) to preserve the nascent polymer (7,47,48). This sensitive immunofluorescence-based assay provides an indirect measure of the level of chromosomal SSBs, analogous to the measurement of γH2AX as a marker of DSBs. Indeed, incubation for short periods with PARG inhibitor uncovered a significant increase in the level of nuclear ADP-ribose in control 1BR fibroblasts following treatment with 2 Gy of IR, and as expected this level was much higher in AOA-XRCC1 patient-derived fibroblasts in which SSBR is known to be reduced (7) (Figure 3A). More importantly, ADP-ribose was also greatly elevated in the AOA4/MCSZ patient fibroblasts, indicative of a similar defect in SSBR in these cells (Figure 3A). We also measured the level of DSBs in these experiments in parallel, but did not detect a difference between control and PNKP patient fibroblasts (Supplementary Figure S2A). Notably, we also detected a reduced rate of SSBR in both AOA4/MCSZ and MCSZ-I patient fibroblasts in experiments in which we employed a 10-fold higher dose of IR (20 Gy), to measure levels of poly(ADP-ribose) in the absence of PARG inhibitor (Supplementary Figure S2B).

To confirm the defect in SSBR in PNKP patient-derived fibroblasts, we also quantified DNA strand breaks following IR directly, using alkaline comet assays. Whilst this assay detects both SSBs and DSBs, >95% of the DNA strand breaks induced by IR are SSBs (49). Importantly, in agreement with the nuclear ADP-ribose assay, the rate at which SSBs declined following 20 Gy of IR was significantly slower both in XRCC1 and PNKP patient-derived fibroblasts, when compared to the 1BR control (Figure 3B). Finally, we also measured the induction and repair of SSBs induced by the abortive activity of TOP1, following treatment with CPT. Importantly, SSBs accumulated to a much higher level in both of the PNKP patient-derived fibroblasts during incubation with CPT, when compared to 1BR control cells, as measured by either nuclear ADP-ribose levels or by alkaline comet assays (Figure 3C and D). We conclude from these experiments that whilst AOA4/MCSZ and MCSZ-I cells exhibit normal rates of DSBR, they possess reduced rates of SSBR.

### Requirement for the DNA 5′-kinase, DNA 3′-phosphatase, and FHA Domains of PNKP for SSBR

**Figure 4.**
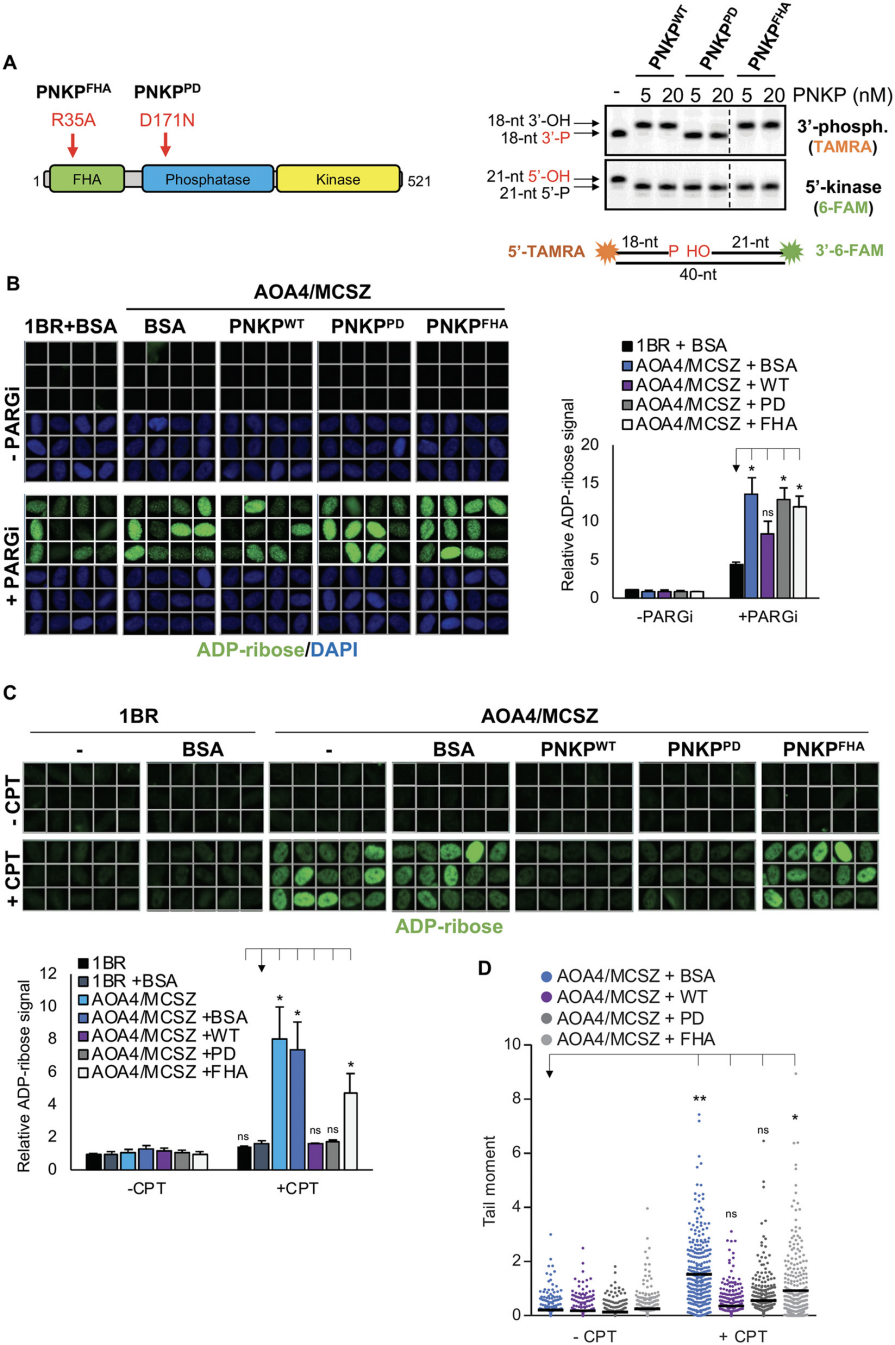
Importance of the PNKP DNA 5′-kinase, 3′-phosphatase, and FHA Domains for SSBR. (**A**) Cartoon (*left*) and enzymatic activity (*right*) of the recombinant PNKP proteins employed here for complementation experiments. For activity assays, a 5′-TAMRA- and 3′-6-FAM- dual-labelled oligonucleotide duplex (shown *bottom*) harbouring a SSB with both 3′-phosphate and 5′-hydroxyl termini was incubated for 10 min with the indicated purified recombinant PNKP proteins prior to fractionation by denaturing PAGE. The positions of the TAMRA-labelled 3′-phosphatase substrate (‘18-nt 3′-P’), 6-FAM-labelled 5′-kinase substrate (‘21-nt 5′-OH’), and the products of their processing by PNKP 3′-phosphatase activity (‘18-nt 3′-OH’) and 5′-kinase activity (‘21-nt 5′-P’), respectively, are shown. (**B**) Representative scanR images (*left*) and quantification (*right*) of nuclear ADP-ribose in control fibroblasts (1BR) and AOA4/MCSZ patient-derived fibroblasts 30 min following ionizing radiation (2 Gy) in the absence or presence of 10 μM PARG inhibitor (PARGi) as indicated. PARGi was added 30 min prior to irradiation. Cells were transfected with BSA or the indicated purified wild-type (PNKP^WT^) or mutant (PNKP^PD^, PNKP^FHA^) PNKP proteins 18 h prior to irradiation. Data are normalized to DMSO-treated 1BR fibroblasts transfected with BSA and are the mean (±SEM) of three independent experiments. Statistically significant differences were determined by one-tailed *t*-test (*P < 0.05; *ns*, not significant). (**C**) Representative scanR images (*top*) and quantification (*bottom*) of nuclear ADP-ribose in the indicated cell lines after 45 min incubation with DMSO vehicle or 10 μM camptothecin (CPT). Cells were transfected in the presence of the indicated proteins 18 h prior to CPT treatment. Data are as described in *(B)*. (**D**) DNA strand breaks quantified by alkaline comet assays in the indicated cell lines following transfection with BSA or the indicated recombinant PNKP proteins and treated 18 h later with DMSO vehicle or 10 μM CPT for 60 min. Data are the individual comet tail moments of 300 cells per sample combined from three independent experiments. The horizontal bars show the mean tail moment. Statistically significant differences were determined using the means from the three independent experiments (100 cells/sample by one-tailed *t*-test (*P < 0.05; **P < 0.01; *ns*, not significant).

To examine which PNKP activities are responsible for the SSBR defect in PNKP-mutated disease we conducted complementation experiments, by transfecting purified wild type and mutant recombinant PNKP proteins into AOA4/MCSZ fibroblasts. AOA4/MCSZ fibroblasts were employed because they possess the least amount of residual DNA 3′-phosphatase activity and completely lack residual DNA 5′-kinase activity. As expected, recombinant wild type histidine-tagged PNKP (His-PNKP^WT^) and His-PNKP^FHA^ harbouring a mutated FHA domain possessed both DNA 5′-kinase activity and DNA 3′-phosphatase activity, whereas His-PNKP^PD^ harbouring a mutated 3′-phosphatase domain lacked the latter (Figure 4A). We also generated recombinant PNKP protein containing a point mutation (K378A) that greatly reduces or ablates DNA kinase activity, but this protein was relatively unstable when transfected into cells and so was not utilized further (unpublished observations).

Whereas transfection with His-PNKP^WT^ partially complemented the SSBR defect in AOA4/MCSZ patient fibroblasts following IR, as measured by a decrease in the level of nuclear ADP-ribose 30 min following irradiation, transfection with His-PNKP^PD^ failed to do so (Figure 4B). This is consistent with the presence of 3′-phosphate at ∼70% of oxidative DNA breaks (14). We also examined which PNKP activities were required to correct the defect in repair of TOP1-induced SSBs, since this type of SSB possess both 3′-phosphate and 5′-hydroxyl termini (15). Surprisingly, both His-PNKP^WT^ and His-PNKP^PD^ completely suppressed the elevated accumulation of TOP1-induced SSBs in AOA4/MCSZ patient fibroblasts following CPT treatment, as measured both by levels of nuclear ADP-ribose and by alkaline comet assays (Figure 4C and D). Similar results were obtained if we employed pEYFP-tagged PNKP cDNA expression constructs, instead of recombinant protein (Supplementary Figure S3A). This result indicates that only the DNA 5′-kinase activity of PNKP is rate limiting for rapid repair of TOP1-induced SSBs in AOA4/MCSZ fibroblasts. Notably, recombinant His-PNKP^FHA^ harbouring a mutated FHA domain failed to restore full repair capacity in AOA4/MCSZ fibroblasts following either IR or CPT, confirming that the recruitment of PNKP by XRCC1 is important for SSBR following either oxidative stress or abortive TOP1 activity. Note that the inability of transfected His-PNKP^FHA^ to restore SSBR was not due to differences in the level of transfected protein, because protein extracts prepared from cells transfected with either His-PNKP^WT^ or His-PNKP^FHA^ possessed similar levels of PNKP biochemical activity, as determined by their ability to repair of an oligonucleotide substrate containing both 5′-hydroxyl and 3′-phosphate termini (Supplementary Figure S3B). Together, these data indicate a collective requirement for all three domains of PNKP for efficient repair of the range of SSBs that arise in cells.

### SSBR functionality and the severity of PNKP-associated disease

**Figure 5.**
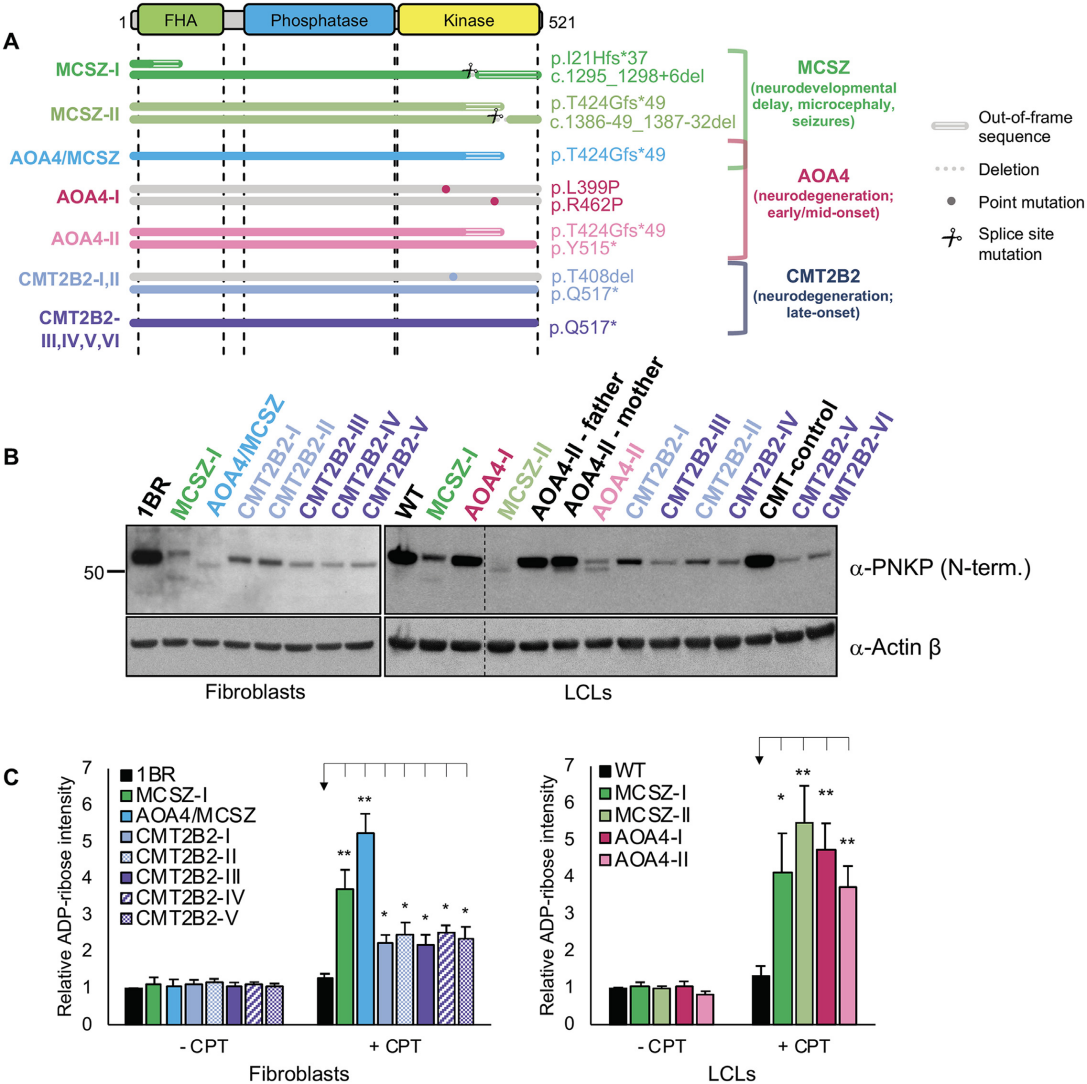
PNKP functionality and disease severity. (**A**) Cartoon depicting the location and type of PNKP mutations present in the MCSZ, AOA4 and CMT2B2 patient-derived cell lines employed in this study. The details of the mutant alleles and the relevant citations are described in the materials and methods. For homozygous mutations only one allele is depicted. (**B**) Western blot showing PNKP protein levels in the indicated control and PNKP patient-derived fibroblasts (*left*) and LCLs (*right*). β actin was employed as a loading control. (**C**) Quantification of nuclear ADP-ribose in the indicated control and PNKP patient-derived cells after 45 min incubation with DMSO vehicle or 10 μM camptothecin (CPT). Data are normalized to DMSO-treated WT cells and are the mean (±SEM) of four independent experiments. Statistically significant differences were determined by one-tailed *t*-test (**P < 0.01; *P < 0.05). Representative scanR images are shown in Supplementary Figure S4B.

Next, we examined whether the severity of PNKP-associated disease is related to the level of residual PNKP protein and/or activity during SSBR. To address this, we first compared the level of PNKP protein in cells derived from individuals with MCSZ with those from individuals with AOA4 or CMT2B2; the milder forms of PNKP-mutated disease (Figure 5A). Whilst, PNKP protein levels were greatly reduced in most of the patient-derived LCLs and fibroblasts, there was no correlation between the level of residual PNKP protein and pathological severity (Figure 5B). For example, the level of PNKP protein in the CMT2B2 cell lines was generally no higher than in the AOA4-I and MCSZ-I cell lines, despite the far milder pathology associated with the former. Similarly, we did not observe any strong correlation between disease severity and the level of residual DNA 5′-kinase or DNA 3′-phosphatase activity (Supplementary Figure S4A). For example, the level of residual 3′-phosphatase activity in the CMT2B2 cell lines was generally no higher than in cell lines from the more severe diseases AOA4 and MCSZ, and the level of 5′-kinase activity in MCSZ-I was higher than in cell lines from the less severe diseases AOA4 and CMT2B2. Consequently, we employed the sensitive nuclear ADP-ribose assay to examine whether disease severity correlated with the overall efficiency of SSBR in the different patient-derived cell lines. Indeed, we detected elevated levels of ADP-ribose in all of the patient-derived cell lines when compared to control cells following treatment with CPT, and the level of CPT-induced nuclear ADP-ribose in MCSZ and AOA4 cell lines was higher than in cell lines from the far less severe disease, CMT2B2 (Figure 5C and Supplementary Figure S4B). Collectively, these data implicate reduced rates of SSBR as a cause of PNKP-associated disease, and suggest that the extent of this reduction is a determinant of disease severity.

## DISCUSSION

### Reduced rates of SSBR, but not DSBR, in PNKP-mutated disease

Mutations in PNKP result in several diseases spanning a spectrum of neurological pathology, from neurodevelopmental dysfunction in microcephaly with early-onset seizures (MCSZ) through to moderate or mild/late-onset neurodegeneration in AOA4 and CMT2B2, respectively. A possible explanation why mutations in PNKP might cause both neurodegeneration and neurodevelopmental dysfunction was the putative role for this protein in the repair of both SSBs and DSBs. Surprisingly, however, we found here that primary fibroblasts derived from individuals with MCSZ or with combined AOA4/MCSZ exhibit normal rates of DSBR following treatment with either ionising radiation (IR) or camptothecin (CPT); physiologically relevant sources of DSBs harbouring the 5′-hydroxyl and/or 3′-phosphate termini that are substrates for PNKP (15). The lack of a significant DSBR defect in PNKP-mutated cells was surprising, because PNKP interacts with the critical NHEJ protein XRCC4 (19,21,50) and has been reported to be required for efficient NHEJ both *in vitro* and in cells (19,20,42,51). It is unlikely that this result reflects a limitation of our γH2AX assay, because we readily detected the expected defect in DSBR if the cells were incubated with an inhibitor of the critical NHEJ protein, DNA-PKcs. Whilst we cannot rule out that the pathogenic mutations in PNKP result in measurable defects in DSBR specifically in neurones this seems unlikely, because the defect in NHEJ in similar microcephalic diseases resulting from mutations in XRCC4, XLF/Cernnunos, or DNA ligase IV is readily detected using patient-derived fibroblasts and assays of the type employed here (38,40,52). One possible explanation for our results is that the residual PNKP activity that is present in the patient-derived fibroblasts is sufficient to maintain DSBR proficiency. Perhaps consistent with this idea, we noted that PNKP siRNA slightly increased the level of residual γH2AX in AOA4/MCSZ cells following IR, albeit not statistically significantly. Alternatively, perhaps other DNA 3′-end processing proteins can substitute for PNKP during NHEJ (53). Irrespective of why DSBR was largely normal in the PNKP patient-derived fibroblasts employed here, our experiments suggest that a defect in DSBR is unlikely to be the cause of PNKP-mutated disease.

In contrast to DSBR, the proficiency of SSBR in MCSZ patient-derived fibroblasts was greatly reduced following either IR or CPT. This is consistent with our previous analyses, in which we detected similar defects in SSBR in lymphoblastoid cell lines derived from different MCSZ patients (54). The defect in SSBR detected here was apparent whether we quantified SSBs directly by alkaline comet assays or indirectly by measuring levels of nuclear ADP-ribose. The additional use of the nuclear ADP-ribose assay was important, because the greater sensitivity of this assay allowed us to measure SSBs at the same low dose of IR as that employed for measuring DSBs (2 Gy). In contrast to cell lines derived from patients with MCSZ however, we failed to detect any defect in SSBR following IR in the less severe diseases AOA4 or CMT2B2 (unpublished observations). However, we did detect a defect in SSBR in AOA4 and CMT2B2 cell lines following treatment with CPT, using this assay. Moreover, the defect in CMT2B2 cells following CPT was less than that detected in either MCSZ or AOA4 cell lines, consistent with CMT2B2 being pathologically the mildest of these diseases. Collectively, these experiments implicate slower rates of SSBR as a cause of PNKP-mutated disease, and suggest that the severity of this disease is related to the level of SSBR capacity.

### MCSZ and reduced DNA 3′-phosphatase functionality

It is currently unclear which of the two enzymatic activities of PNKP contribute most to the neuroprotective role of this protein during SSBR. To address this, we compared patient-derived cell lines from the different PNKP-mutated diseases for levels of PNKP protein and its two biochemical activities. However, we failed to detect a correlation between disease severity and any of these parameters individually. It is possible that other factors contribute to disease severity, such as an additive impact of the reduction in the two PNKP catalytic activities or as yet unidentified differences in other DNA repair pathways. Alternatively, perhaps the level of residual PNKP activity detected *in vitro* fails to fully reflect the functionality of the mutant protein in cells. Indeed, consistent with this idea, only cell lines from patients with MCSZ exhibited a reduced ability to repair chromosomal SSBs induced by IR [this work and (54)]. Since IR induces SSBs with 3′-phosphate termini this observation suggests that the DNA phosphatase functionality of PNKP is lower in MCSZ cells than in AOA4 and CMT2B2 cells, despite the lack of a detectable difference in their DNA phosphatase activity in cell extracts in vitro. One possible explanation for this discrepancy is that our biochemical assays are not sufficiently sensitive to detect pathologically relevant differences between the DNA phosphatase activity of the different patient cell lines. Alternatively, perhaps the large truncation mutations that are common in MCSZ affect not only the catalytic activity of PNKP but also its ability to be recruited and and/or stimulated by XRCC1. This latter idea may explain why MCSZ-I cells, which retain relatively high levels of residual PNKP activity but which harbour a mutated FHA domain, possess a defect in SSBR following IR. However, irrespective of why there is a discrepancy between our biochemical and cellular assays, the observation that only MCSZ cell lines exhibit a defect in repair of IR-induced SSBs implicates reduced PNKP-dependent DNA phosphatase activity in the development of MCSZ.

In contrast to reduced DNA phosphatase activity, an involvement of reduced DNA kinase activity in causing MCSZ is less likely. For example, the AOA4 cell lines (AOA4-I and AOA4-II) exhibited levels of SSBR following CPT as low as those in MCSZ cells. Since we found here that reduced SSBR following CPT is a measure of reduced 5′-DNA kinase function this suggests that reduced DNA kinase activity does not, by itself at least, cause MCSZ. This conclusion is also consistent with our previous observation that the PNKP point mutation E326K, which results in MCSZ, reduces the proficiency of SSBR following IR but not CPT (54).

### Neurodegeneration and reduced DNA 5′-kinase functionality

In contrast to the neurodevelopmental pathology that typifies MCSZ, it seems unlikely that reduced DNA 3′-phosphatase functionality can account for the neurodegenerative pathology that typifies AOA4 and CMT2B2. For example, as discussed above, whilst we readily detected a defect in the repair of IR-induced SSBs with 3′-phosphate termini in MCSZ cells, we failed to do so in cell lines from either AOA4 or CMT2B2; the PNKP-mutated diseases that are associated with neurodegeneration (data not shown). Indeed, our data suggest that it is reduced DNA 5′-kinase activity that is the major contributor and/or cause of the neurodegeneration in PNKP-mutated disease, because we detected reduced SSBR in all of our AOA4 and CMT2B2 cell lines following treatment with CPT, which as discussed above is a measure of DNA 5′-kinase functionality. Moreover, the extent of this SSBR defect was greater in AOA4 than in CMT2B2 cells, consistent with the relative severity and/or age of onset of neurodegeneration in these diseases. If reduced DNA 5′-kinase is a cause of neurodegeneration in PNKP-mutated disease then this pathology should also be present in most patients with MCSZ, most of which based on our analyses harbor residual DNA kinase activity and rates of CPT-induced SSBR as low or lower than AOA4 and CMT2B2. Indeed, while cerebellar atrophy and ataxia was not initially reported as a feature of MCSZ, more recent case reports have reported this pathology (26–30).

Finally, our data have implications concerning the identity of the endogenous SSBs that trigger neurological dysfunction in PNKP-mutated disease. For example, if reduced 3′-phosphatase is indeed the cause of neurodevelopmental dysfunction these data implicate SSBs arising from oxidative stress in MCSZ, because this is a major source of DNA breaks with 3′-phosphate termini. Similarly, if reduced DNA 5′-kinase activity is a cause of cerebellar ataxia and neurodegeneration in PNKP-mutated disease our results implicate SSBs arising from abortive TOP1 activity in AOA4 and CMT2B2, because this is a major source of DNA breaks with 5′-hydroxyl termini. The latter observation is consistent with the molecular defect in the related SSBR-defective neurodegenerative disease, SCAN1, which is similarly required for the repair of SSBs induced by abortive TOP1 activity (8,55). It is also consistent with recent reports that the repair of TOP1-induced SSBs is reduced in ataxia telangiectasia; the archetypal neurodegenerative disease associated with defects in DNA strand break repair (56,57).

In summary, our data implicate reduced SSBR, but not DSBR, as a cause of PNKP-mutated disease, and suggest that the pathological severity of this disease is determined by the nature and extent of this reduction. Moreover, our data also highlight the two enzymatic activities of PNKP in different aspects of the disease pathology, with reduced DNA phosphatase and DNA kinase activity correlating with neurodevelopmental dysfunction and neurodegeneration, respectively.

## Supplementary Material

gkaa489_Supplemental_File
