# Supplementary material for: Pathological mutations in PNKP trigger defects in DNA single-strand break repair but not DNA double-strand break repair
Source: Nucleic Acids Res. 2020 Jun 6;48(12):6672–84. doi: 10.1093/nar/gkaa489 (PMC7337934; doi:10.1093/nar/gkaa489)
Supplement: gkaa489_Supplemental_File [file gkaa489_supplemental_file.pdf]

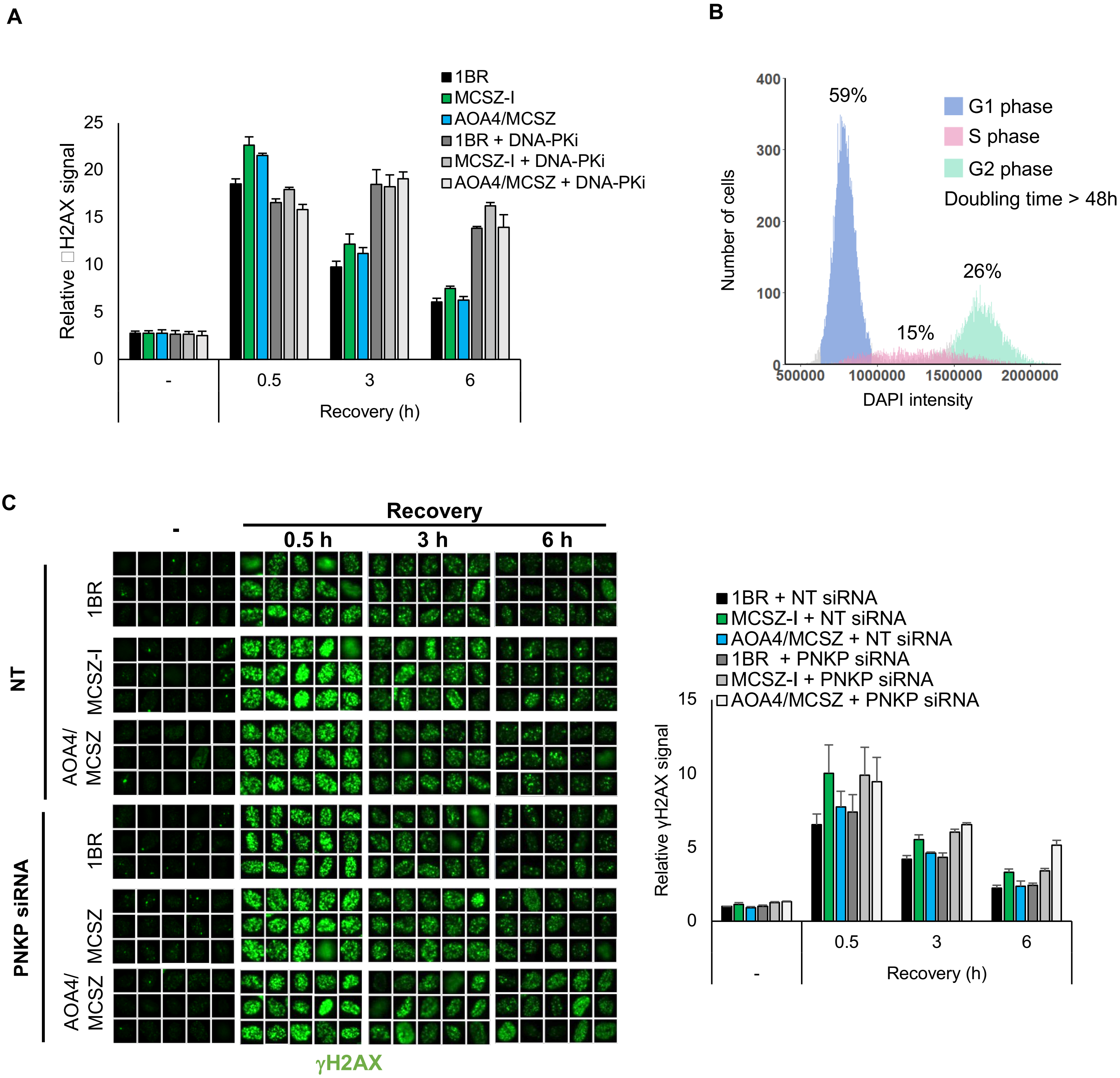

Figure S1. Detection of DSBs in control and PNKP patient-derived fibroblasts before and after DNA damage (related to Figure 2). (A) Quantification of total γH2AX intensities from the experiment in *Figure 2A* (which shows representative images and normalized data).**(B)** A cell cycle profile of a representative scanR experiment from *Figure 2A*. Approximately 59% of fibroblasts were at the time of analysis in G1 phase (blue), 15% in S phase (pink) and 26 % in G2 phase (green) and of the cell cycle. Cells which were excluded from the analysis are shown in grey. The cell cycle profile of all cell lines was similar and their doubling time was >48h. **(C)** Representative scanR images (*left*) and quantification of total γH2AX (*right*) from the experiment in *Figure 2C* (which shows the normalized data).

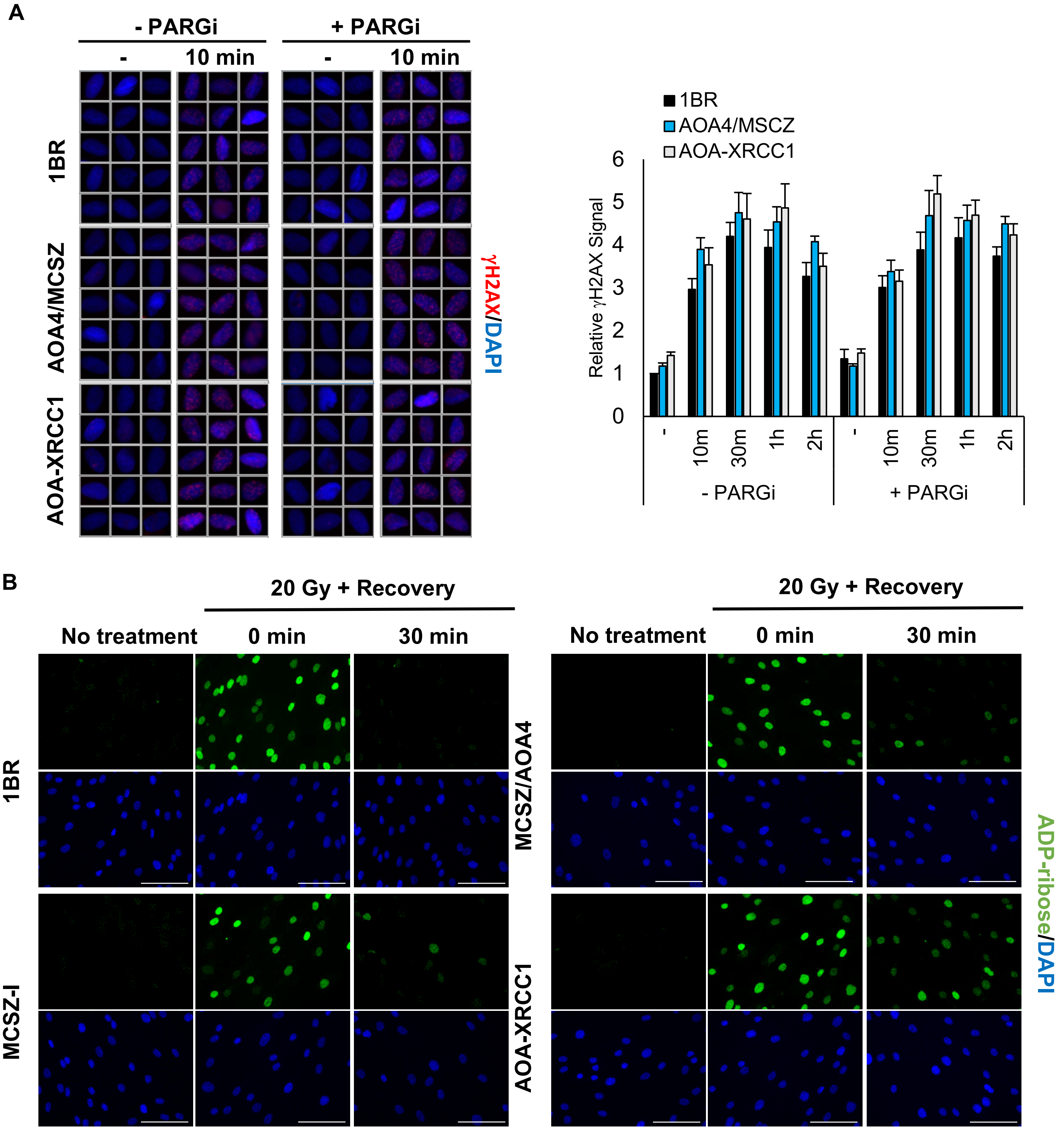

Figure S2. Detection of DSBs in control and PNKP patient-derived fibroblasts before and after DNA damage (related to Figure 3). (A) Representative scanR images (*left*) and quantification (*right*) of γH2AX immunostaining conducted in the experiments in *Figure 3A*. **(B)** Representative images of nuclear ADP-ribose levels in control (1BR) and PNKP patient-derived (AOA4/MCSZ) or XRCC1 patient-derived (AOA-XRCC1) fibroblasts before and 30 min after treatment with ionizing radiation (20 Gy).

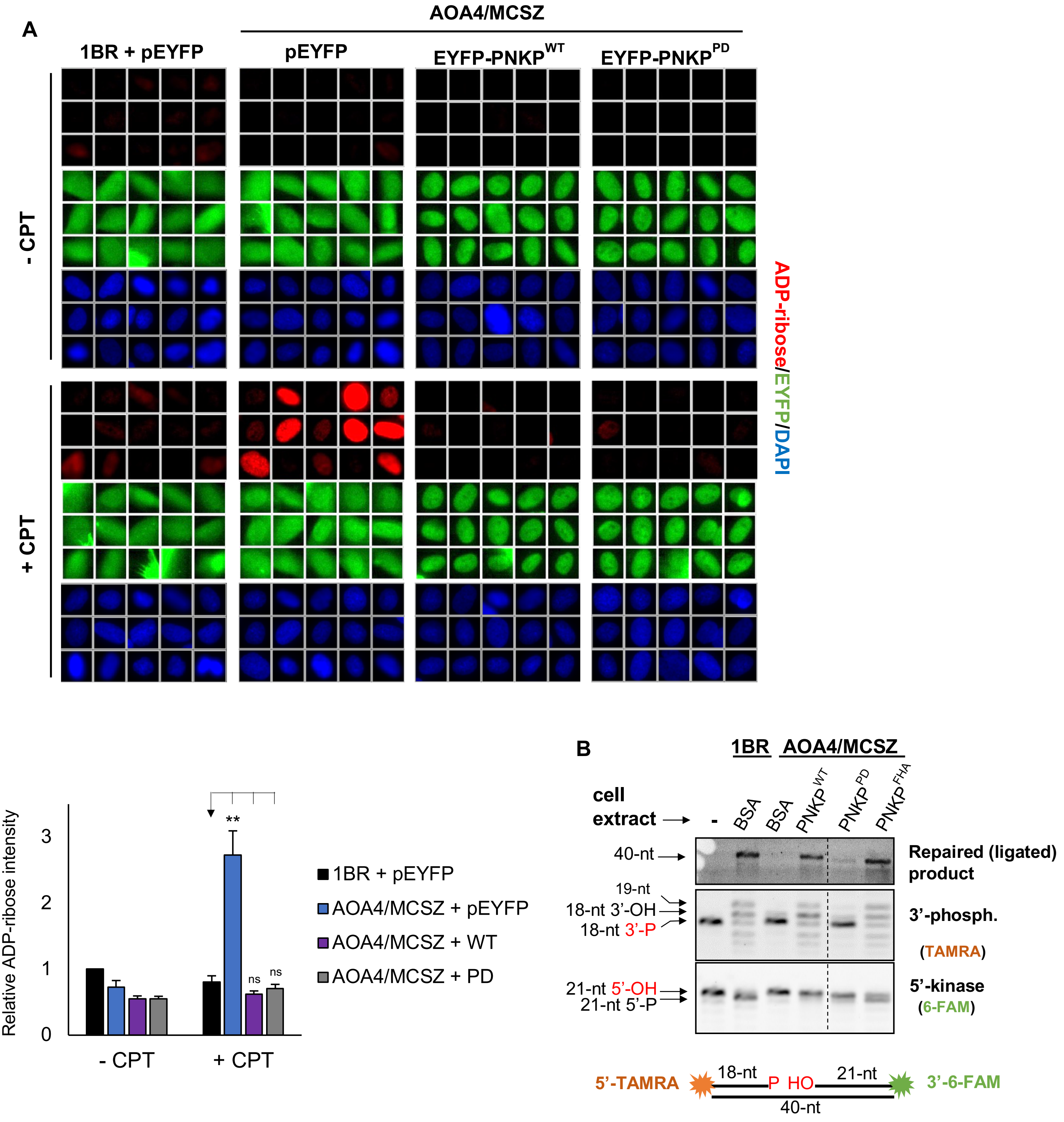

Figure S3. The DNA 5'-kinase activity of PNKP complements the SSBR defect at Top1-induced SSBs in PNKP patient-derived fibroblasts (related to Figure 4). (A) Representative scanR images (*top*) and quantification of nuclear ADP-ribose (*bottom*) in control 1BR and AOA4/MCSZ patient-derived fibroblasts transfected with empty pEYFP-C1 or pEYFP-C1 encoding wild type ($PNKP^{WT}$) or phosphatase-dead ($PNKP^{PD}$) cDNA. Cells were transfected 48 h before a 45 min treatment with DMSO vehicle or 10 μM camptothecin (CPT). Data for EYFP-positive cells are shown and are the mean (±SEM) of four independent experiments. Statistical analysis (one-tailed *t*-test) comparing CPT-treated 1BR and AOA4/MCSZ fibroblasts is indicated (**P < 0.01; *ns*, not significant). **(B)** PNKP activity in wild type (1BR) and AOA4/MCSZ patient fibroblasts electroporated with BSA control or the indicated purified recombinant PNKP proteins employed in *Figure 4A*. For activity assays, a 5'-TAMRA- and 3'-6-FAM- dual-labelled oligonucleotide duplex (*bottom*) harbouring a SSB with both 3'-phosphate and 5'-hydroxyl termini was incubated for 60 min at 37°C with the indicated cell extracts (15 μg total protein) prior to fractionation by denaturing PAGE. *Arrows* indicate the positions of the TAMRA-labelled 3'-phosphatase substrate ("18-nt 3'-P"), FAM-labelled 5'-kinase substrate ("21-nt 5'-OH") and intermediates of their repair resulting from 3'-phosphatase activity ("18-nt 3'-OH"), DNA kinase activity ("21-nt 5'-P"), DNA gap filling ("19-mer"), and DNA ligation ("40-nt").

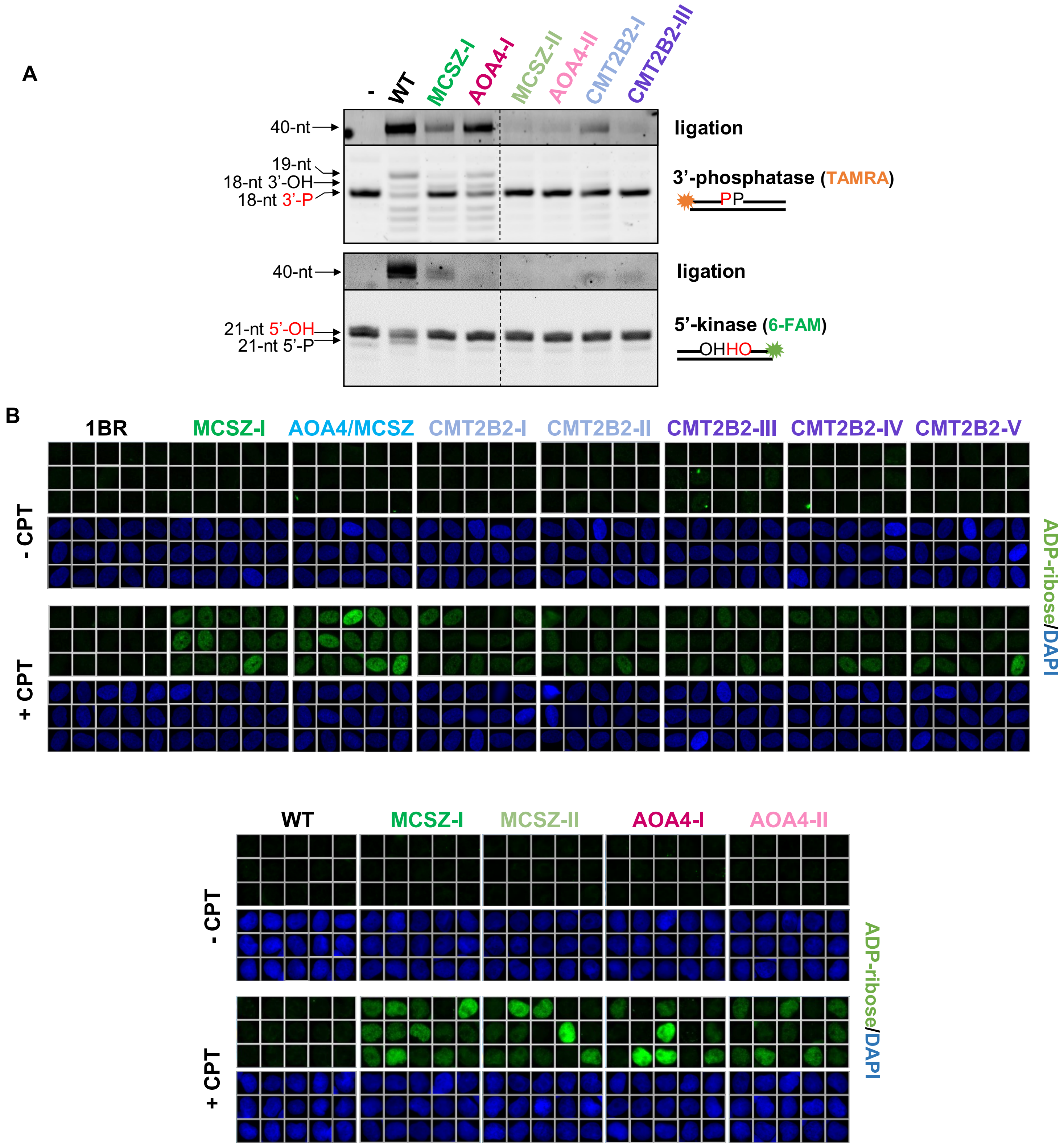

Figure S4. PNKP functionality and disease severity (related to Figure 5). (A) DNA 3'-phosphatase (*top*) and DNA 5'-kinase (*bottom*) activity in cell extracts prepared from the indicated control and patient-derived LCLs. TAMRA- or 6-FAM-labelled PNKP oligonucleotide duplex harbouring a SSB with a 3'-phosphate or 5'-hydroxyl terminus, respectively, was incubated with the indicated cell extracts (25 μg total protein) for 10 min at 37°C prior to fractionation by denaturing PAGE. 3'-phosphatase, 5'-kinase. *Arrows* indicate the positions of the TAMRA-labelled 3'-phosphatase substrate ("18-nt 3'-P"), 6-FAM-labelled 5'-kinase substrate ("21-nt 5'-OH"), and intermediates of their repair resulting from 3'-phosphatase activity ("18-nt 3'-OH"), 5'-kinase activity ("21-nt 5'-P"), DNA gap filling ("19-mer"), and DNA ligation ("40-nt"). **(B)** Representative scanR images of single-cell galleries from the experiments in *Figure 5C*.
